# Retraction: MicroRNA-595 sensitizes ovarian cancer cells to cisplatin by targeting ABCB1

**DOI:** 10.18632/oncotarget.28838

**Published:** 2026-04-24

**Authors:** Songyu Tian, Mingyue Zhang, Xiuwei Chen, Yunduo Liu, Ge Lou

**Affiliations:** ^1^Department of Gynecology Oncology, Cancer Hospital of Harbin Medical University, Harin, 150081, Heilongjiang, China; ^2^Department of Anaesthesiology, Cancer Hospital of Harbin Medical University, Harin, 150081, Heilongjiang, China

**This article has been retracted**: Oncotarget’s Image Forensics investigation of this paper has revealed multiple instances of image duplication and overlap with unrelated papers. Specifically:

Figure 3: The Transwell assay image in Figure 3I was previously published in Figure 5D of unrelated paper [1] and Figure 3C of [2]. Additionally, the image from Figure 3J was found in Figure 3C of [2].
Figure 6: The Transwell assay image in Figure 6M was found in Figure 3A of the earlier published unrelated paper [2]. Western Blot images from Figure 6B were found in Figure 4G and Figure 6C of the concurrently published articles [3, 4], respectively, and in Figures 3A and 6E of the 2017 article [5], which has already been retracted.

The authors did not respond to requests to comment on these concerns or provide the original data. Consequently, the Editorial decision has been made to retract this paper. Oncotarget has reached out to all authors to confirm this retraction, but received no response.

Original article: Oncotarget. 2016; 7:87091–87099. 87091-87099. https://doi.org/10.18632/oncotarget.13526
